# Elements of Medicine

**Published:** 1859-08

**Authors:** 


ART. VIII.—*Elements of Medicine. A Compendious View of Pathology and Therapeutics; or, the History and Treatment of Diseases.* By SAMUEL HENRY DICKSON, M.D., LL.D., Professor of the Practice of Physic in Jefferson Medical College, Philadelphia; formerly Professor of the Institutes and Practice of Medicine in the Medical College of the State of South Carolina, and of the Theory and Practice of Medicine in the New York University. Second Edition. Revised. Philadelphia: Blanchard & Lea, 1859.

Since the appearance of Dickson's Elements of Medicine, two years ago the author has been appointed to the Chair of Practice in Jefferson Medical College, a position which would almost inevitably have insured a speedy sale of his work. The immense number of students which yearly congregate in the lecture-rooms of the Jefferson, go to their homes armed with copies of most of the productions of the Faculty, all of whom are authors more or less voluminous. When this volume first appeared, the reviewer in this Journal made certain strictures, which we cannot but indorse. These were in regard to the imperfect manner in which some of the most important diseases are considered. In examining the new edition, we cannot see that these defects have been remedied, and the work is only enlarged by a few pages. That a treatise on practice which is limited to seven hundred and sixty-two pages, can be a sufficient authority for the young practitioner, or even complete enough to serve as a text-book for the student, seems to us out of the question. A surgery is usually much larger; and we are sure that the domain of practical medicine, and its importance to the greater number of practitioners, is much greater than that of surgery. In our judgment, Dr. Dickson has undertaken an impossibility, in endeavoring to compress the subject of practice into a work no larger than the one before us; and we are confident that the student or practitioner who relies entirely upon it as a guide, will often be misled. We are not at liberty to make the foregoing criticisms without citing at least one illustration. Turning to page 356, we find what is said of valvular disease of the heart. Every one will admit the importance and frequency of these disorders; yet to the diagnosis, prognosis, pathology, and treatment of all varieties of valvular lesion, we have devoted the space of precisely *two pages*. Can it be possible that such a consideration of this subject should be of the slightest value? Though the work before us is not without some good qualities, we cannot, as a conscientious journalist, pass over such glaring imperfections as this.

